# Efficient Non-Viral Ocular Gene Transfer with Compacted DNA Nanoparticles

**DOI:** 10.1371/journal.pone.0000038

**Published:** 2006-12-20

**Authors:** Rafal Farjo, Jeff Skaggs, Alexander B. Quiambao, Mark J. Cooper, Muna I. Naash

**Affiliations:** 1 Department of Cell Biology, University of Oklahoma Health Sciences Center Oklahoma City, Oklahoma, United States of America; 2 Copernicus Therapeutics, Inc. Cleveland, Ohio, United States of America; Baylor College of Medicine, United States of America

## Abstract

**Background:**

The eye is an excellent candidate for gene therapy as it is immune privileged and much of the disease-causing genetics are well understood. Towards this goal, we evaluated the efficiency of compacted DNA nanoparticles as a system for non-viral gene transfer to ocular tissues. The compacted DNA nanoparticles examined here have been shown to be safe and effective in a human clinical trial, have no theoretical limitation on plasmid size, do not provoke immune responses, and can be highly concentrated.

**Methods and Findings:**

Here we show that these nanoparticles can be targeted to different tissues within the eye by varying the site of injection. Almost all cell types of the eye were capable of transfection by the nanoparticle and produced robust levels of gene expression that were dose-dependent. Most impressively, subretinal delivery of these nanoparticles transfected nearly all of the photoreceptor population and produced expression levels almost equal to that of rod opsin, the highest expressed gene in the retina.

**Conclusions:**

As no deleterious effects on retinal function were observed, this treatment strategy appears to be clinically viable and provides a highly efficient non-viral technology to safely deliver and express nucleic acids in the retina and other ocular tissues.

## Introduction

The eye is comprised of several specialized tissues that work together to initiate visual perception in response to photons of light. Any insult to these tissues results in a consequence to vision and an impact on the quality of life for the patient. Both environmental trauma and genetic disorders can cause varying degrees of ocular diseases. Current therapies for ocular disorders are often surgically-based or topical treatments however they often fail to correct the underlying genetic deficit. As the eye is easily accessible and immune-privileged, the use of gene transfer is an attractive therapeutic option for numerous forms of blinding disorders.

Many disease-causing mutations and their contribution to the pathogenesis of ocular diseases including cataracts, glaucoma, and retinitis pigmentosa have been well characterized [1]–[5]. In addition, several treatment strategies for overcoming these genetic deficits have been attempted and proven in tissue culture and various animal models [6]–[10]. Many attempts to rescue genetic deficits with viral gene therapy have proven effective in the eye [11]–[15]. Perhaps the most encouraging trial has occurred in Briard dogs harboring a naturally occurring mutation in the RPE65 gene, which causes visual impairment similar to Early Onset Severe Retinal Dystrophy in humans [16], [17]. Bennett and colleagues used adeno-associated virus to express the RPE65 cDNA which restored retinal function and has successfully persisted over 3 years [18]–[20]. Although viral vectors also have been successful in alleviating hereditary retinal degeneration in mice [12], [21], they can be limited by cell tropism, the size of the expression cassette to be transferred, and host immunity to repeat infections [22], [23]. Additionally, concerns regarding the safety of using viral vectors in human patients have been raised and some trials have resulted in oncogenesis or even mortality [22], [24]–[26].

Towards the mission of the NIH roadmap for nanomedicine and to identify a complementary approach for gene transfer, we examined the feasibility of using compacted DNA nanoparticles as an efficacious platform for non-viral gene transfer to the eye. These nanoparticles consist of a neutrally-charged complex containing a single molecule of plasmid DNA compacted with polyethylene glycol (PEG)-substituted lysine peptides [27]. These complexes are stable in saline and serum, can efficiently transfect post-mitotic airway cells following in vivo delivery, are non-toxic following lung delivery, and can be repetitively dosed without decrement in biologic activity [27]–[29]. The size of the expression cassette does not appear to be a limiting factor as plasmids up to 20 kbp have demonstrated cellular transfection and gene transfer [30]. Varying the counterion at the time of compaction can lead to different 3-dimensional shapes of the nanoparticles which can facilitate the development of customized nanoparticles to transfect a multitude of cell types [31]. Clinical studies also have demonstrated the safety and effectiveness of this system in human subjects [32]. In this study, we demonstrate that the use of compacted DNA nanoparticles is an effective and robust platform for gene transfer to various tissues of the eye. Varying the site of injection and type of nanoparticle resulted in cell-specific transfection. Furthermore, altering the dose of the injected nanoparticles can allow fine-tuning to the correct level of gene expression needed for the therapeutic gene. This gene delivery approach appears to be an excellent strategy for gene transfer to ocular tissues.

## Methods

### Plasmid

pZEEGFP5.1 (5,147 bp) encodes the enhanced green fluorescent protein cDNA transcriptionally-controlled by the CMV immediate-early promoter and enhancer, as previously described [33].

### Nanoparticle Formulation

DNA nanoparticles were formulated by mixing plasmid DNA with CK_30_PEG10K, a 30-mer lysine peptide with an N-terminal cysteine that is conjugated via a maleimide linkage to 10 kDa polyethylene glycol, as previously described [29]. Nanoparticles were concentrated up to 4 mg/ml of DNA in saline.

### Mice

All experiments and animal maintenance were approved by the local Institutional Animal Care and Use Committee (Oklahoma City, OK, U.S.A.) and conformed to the guidelines on the care and use of animals adopted by the Society for Neuroscience and the Association for Research in Vision and Ophthalmology (Rockville, MD, U.S.A.). Balb/cJ mice were obtained from Jackson Labs (www.jax.org) and used for all experiments.

### Quantitative RT-PCR

qRT-PCR was performed as previously described [34]. Briefly, total RNA was extracted from the tissue of a single eye using Trizol reagent (*Invitrogen Inc.*) and then DNAse treated with RNAse-free-DNAse I (*Promega Inc.*). Incubation of 5 U DNAse/ug RNA was carried out at 37° for 1hr, as these excessive conditions were needed to eliminate any isolated nanoparticles that could skew downstream qRT-PCR analyses. Reaction cleanup was performed using an RNeasy mini kit (*Qiagen Inc.*). Reverse transcription was performed using an oligo-dT primer and Superscript III reverse transcriptase (*Invitrogen Inc.*). To ensure the complete removal of nanoparticles, PCR was performed on all cDNA samples using primers (CMV-F1/CMV-R1) to amplify a region of the CMV promoter as this region would only be amplified due to the presence of contaminating nanoparticle or plasmid DNA. Amplification was only detected in positive control samples of pure nanoparticle DNA, demonstrating sufficient removal of nanoparticle contamination from isolated RNA samples. qRT-PCR was performed in triplicate on each cDNA sample using an iCycler (*Bio-Rad Inc.*) and *Δ*cT values were calculated against the neuronal housekeeping gene hypoxanthine phosphoribosyltransferase (*Hprt*). *Hprt* was assigned an arbitrary expression level of 10,000 and relative gene expression values were calculated by the following calculation: Relative Expression  = 10,000/2^ΔcT^, where *Δ*cT = (*Gene* cT−*Hprt* cT). This was repeated with 4 independent samples for each experimental group and the mean expression value is presented with the standard deviation. Agarose gel electrophoresis and disassociation curve analysis were performed on all PCR products to confirm proper amplification. Primer sequences were: HPRT-F: GCAAACTTTGCTTTCCCTGGTT; HPRT-R: CAAGGGCATATCCAACAACA; CMV-F1: AACGCCAATAGGGACTTTCC; CMV-R1: TATCCACGCCCATTGATGTA; EGFP-F: CACATGAAGCAGCACGACTT; EGFP-R: AGTTCACCTTGATGCCGTTC; RHO-F: TCACCACCACCCTCTACACA; RHO-R: TAGTCAATCCCGCATGAACA; SAG-F: CCCTCTCGTGACTTCTCTGG; SAG-R: CTTCCCCTTCACAAGCTCAG; GNAT1-F: GAGGATGCTGAGAAGGATGC; GNAT1-R: GACATGTCCTTGGGCATTGT; RDS-F:GTTCAAGTGCTGTGGGAACA; RDS-R: CTGTGTGGAGGTAGCGGAGT.

### Fluorescence Microscopy

Tissue fixation and sectioning were performed as previously described [35]. Briefly, eyes from mice at 2-days post-injection were enucleated and fixed in 4% paraformaldehyde/PBS for 16 hr at 4°C prior to embedding in OCT media and subsequent freezing of the block. Tissue sections (10 µm thickness) were cut with a microtome and vectashield with DAPI (*Vector Laboratories Inc.*) was applied prior to coverslipping. Sections were viewed at room temperature with an Axioskop 50 (*Carl Zeiss Inc.*) in the autoexpose mode using a ×20, ×40, or ×63 objective. Images were captured with an Axiocam HR digital camera (*Carl Zeiss Inc.*) using Axiovision 3.1 software (*Carl Zeiss Inc.*). Panels labeled “FITC” represent images captured of native GFP fluorescence in the 500–60 nm wavelength when cryosections were subjected to excitation at 485 nm.

### Immunohistochemistry

Tissue fixation and sectioning were performed as previously described [34]. Briefly, eyes from mice at 2 days post-injection were enucleated and fixed in 4% paraformaldehyde/PBS for 16 h 4°C prior to paraffin embedding. Tissue sections (10 µm thickness) were deparaffinized, rehydrated, and blocked in 5% BSA/PBS for 30 min at room temperature (RT). Slides were briefly washed with PBS and incubated with anti-GFP (Molecular Probes Inc.) at 1∶1000 in 1× BSA/PBS for 2 h at RT. Following a brief washing in PBS, vectashield with DAPI (*Vector Laboratories Inc.*) was applied and the slide was coverslipped. Sections were viewed at room temperature with an Axioskop 50 (*Carl Zeiss Inc.*) in the autoexpose mode using a ×20, ×40, or ×63 objective. Images were captured with an Axiocam HR digital camera (*Carl Zeiss Inc.*) using Axiovision 3.1 software (*Carl Zeiss Inc.*).

### Electroretinography

Subretinal injection of 1 µL Saline (0.9%), naked plasmid (0.6 µg), AC-GFP (0.6 µg), or TFA-GFP (0.6 µg) was performed. At 7 days post-injection, ERG analyses were performed as previously described [34]. For the assessment of scotopic response, a stimulus intensity of 1.89 log cd s m^−2^ was presented to the dark-adapted, dilated eyes in a Ganzfeld (model GS-2000; Nicolet). To evaluate photopic response, animals were light adapted for 5 minutes under a light source of 1.46 log cd m^−2^ intensity, and a strobe flash was presented to the dilated eyes in the Ganzfeld with a stimulus intensity of 1.89 log cd s m^−2^. Significance was determined using one-way analysis of variance (ANOVA) and post-hoc tests using Bonferroni's pair-wise comparisons (Prism, ver. 3.02; GraphPad, San Diego, CA).

### Electron Microscopy

Electron micrographs were prepared using carbon-coated electron microscope grids (*Ted Pella*) and counterstained with uranyl acetate, as previously described [33].

## Results

**Figure 1:**
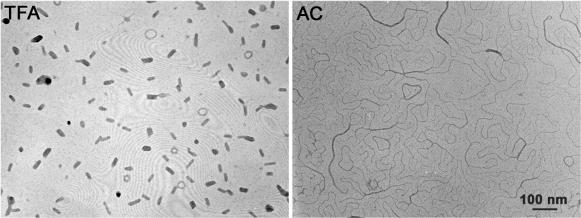
Differential shape of the trifluoroacetate (TFA) and acetate (AC) nanoparticles as visualized by electron microscopy. During compaction of the EGFP expression plasmid, the presence of TFA as the lysine counterion produces ellipsoidal nanoparticles with a minor diameter <18 nm whereas acetate produces rods with a minor diameter <8 nm. Scale bar, 100 nM.

To determine the utility of these nanoparticles in ocular gene therapy, we conducted a set of proof-of-principle experiments using a plasmid to express enhanced green fluorescent protein (EGFP) driven by the cytomegalovirus (CMV) promoter (pZEGFP5.1). We also chose to examine two different nanoparticle formulations generated by the presence of either acetate (AC) or trifluoroacetate (TFA) as the lysine amine counterion at the time of DNA compaction. The TFA particles are ellipsoids and short rods while the AC particles form longer rod-like structures (Fig 1). In both cases, the minor diameter of each particle is less than the 25 nm nuclear membrane pore, facilitating uptake into the nucleus [29]. Furthermore, to target different ocular tissues, we varied the site of ocular injection between the intravitreal space and subretinal space as we hypothesized that the area of delivery may cause different cell types to express EGFP. For every mouse, only one eye was injected while the contra-lateral eye was mock-injected to serve as a control.

**Figure 2:**
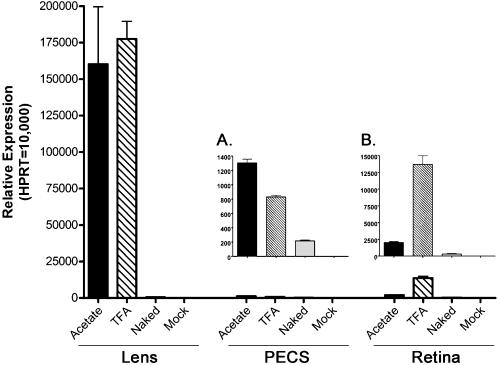
Ocular expression levels of EGFP mRNA after intravitreal injection. At 2 days post-intravitreal injection of 0.6 µg of compacted DNA, the lens, pigment epithelium/choroid/sclera (PECS), and retina were isolated. qRT-PCR was used to assess EGFP mRNA levels and normalized to the neuronal housekeeping gene HPRT. Both the AC-GFP and TFA-GFP nanoparticles produced substantial expression of EGFP in the lens as compared to the naked plasmid control. In the PECS (A) and retina (B), both nanoparticles produced EGFP mRNA at levels several fold higher than naked plasmid. No expression of EGFP was detected in any of the mock-injected samples.

Intravitreal injections of 1 µL of 0.6 µg/µL AC-GFP, TFA-GFP, or naked plasmid DNA were performed in adult Balb/c mice and evaluated by quantitative RT-PCR (qRT-PCR) for EGFP expression at 2 days post-injection. Following euthanasia, the lens and retina were individually separated from the retinal pigment epithelium, choroid, and sclera (PECS). Expression analysis demonstrated that both the AC-GFP and TFA-GFP nanoparticles were capable of promoting high levels of EGFP expression in the lens after intravitreal injection (Fig 2). Substantial expression of EGFP was also detected in the PECS and retina following intravitreal injection, albeit at levels multi-fold lower than that observed in the lens. Delivery of the naked plasmid generated minimal levels of EGFP expression and no expression was detected in the mock injected samples.

**Figure 3:**
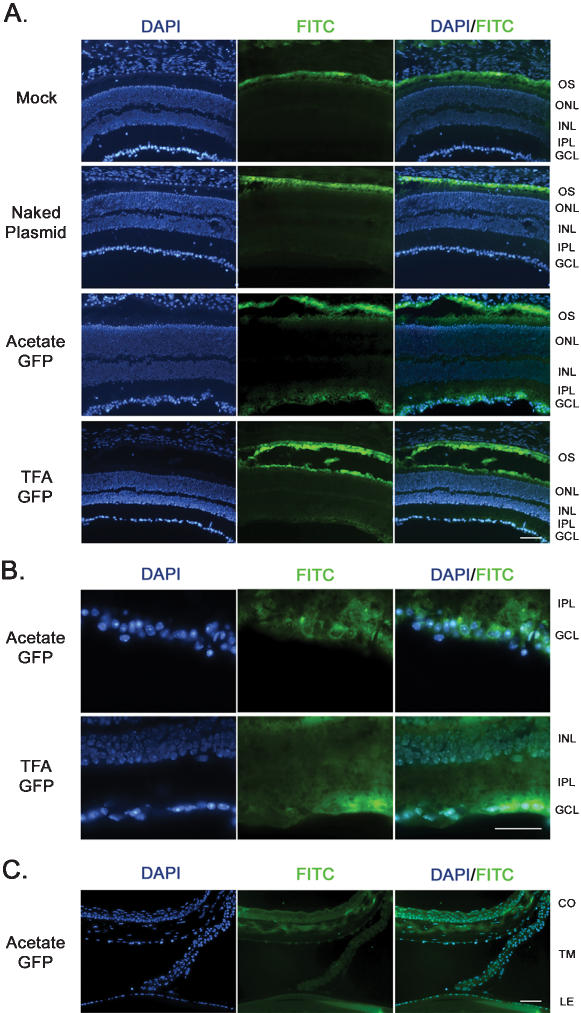
EGFP localization in ocular tissues following intravitreal injection. Direct fluorescence microscopy was utilized to localize EGFP expression in ocular tissues at 2-days post-injection of 0.6 µg of compacted DNA. (**A**) Retinal autofluorescence was present in the outer segment of all samples including mock- and naked plasmid-injected eyes, however EGFP fluorescence was detected in the GCL of both the AC-GFP and TFA-GFP injected eyes. (**B**) Upon examination at higher magnification, EGFP expression was confined to the GCL in the AC-GFP-injected eyes, and present in the GCL and IPL of TFA-GFP-injected eyes. (**C**) Expression of EGFP was also detected in the cornea, trabecular meshwork, and lens following intravitreal injection of both TFA and AC nanoparticles (only AC shown). “FITC” channel imaging was performed as described in the Methods Section. OS: Outer Segment; ONL: Outer Nuclear Layer; INL: Inner Nuclear Layer; IPL: Inner Plexiform Layer; GCL: Ganglion Cell Layer; CO: Cornea; TM: Trabecular Meshwork; LE: Lens. Scale bars, 10 µM.

To determine which cells were transfected after intravitreal injection, direct fluorescence microscopy was performed on ocular cryosections at 2-days post injection. The mock- and naked plasmid-injected eyes showed fluorescence in the FITC channel throughout the outer segment (OS) layer of the retina (Fig 3a). This observation is common in the FITC channel of retinal cryosections as the photoreceptor OSs are packed tightly with the opsin protein which causes this natural autofluorescence [36]–[38]. No other fluorescent signal was observed in the mock- or naked plasmid-injected eyes except in the OS. Examination of the eyes injected with TFA-GFP and AC-GFP nanoparticles showed a fluorescent signal in the inner plexiform layer for TFA-GFP and in retinal ganglion cells (RGCs) for both particles (Fig 3b). Substantial EGFP fluorescence was also detected in the lens, cornea, and trabecular meshwork (Fig 3c), consistent with the qRT-PCR findings.

**Figure 4:**
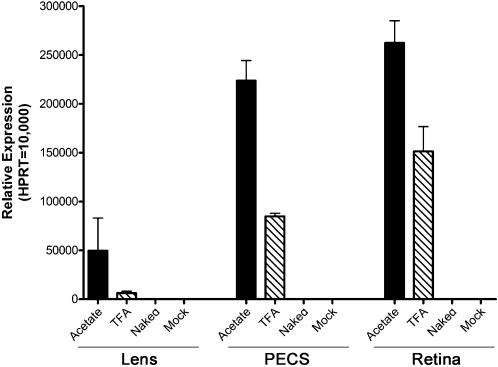
Ocular expression levels of EGFP mRNA after subretinal injection. At 2-days post-subretinal injection of 0.6 µg of compacted DNA, the lens, PECS, and retina were isolated, and qRT-PCR was used to assess EGFP mRNA levels. Both the AC-GFP and TFA-GFP nanoparticles produced substantial expression in the PECS and retina. In both tissues, the AC-GFP nanoparticle generated EGFP mRNA levels nearly 2-fold higher than the TFA-GFP nanoparticle. EGFP expression was also observed in the lens; however there was significant deviation between samples, possibly reflecting the amount of nanoparticle reflux into the vitreous because of back-pressure during syringe withdrawal following some of the subretinal injection procedures.

To target nanoparticle driven expression of EGFP in the photoreceptors and RPE, we delivered 1 µL of AC-GFP, TFA-GFP, or naked plasmid to the retina by a subretinal injection. Our laboratory has previously demonstrated this method is capable of delivering material to over 90% of the photoreceptor cells [39]. After 2-days post-injection, ocular tissues were isolated as described above and examined for EGFP mRNA expression by qRT-PCR (Fig 4). Subretinal injection of AC-GFP and TFA-GFP was extremely effective in producing EGFP expression in the PECS and retina. Levels of EGFP after AC-GFP delivery were 2-fold higher in the PECS and 1.5-fold higher in the retina. Expression of EGFP after subretinal injection of naked plasmid was minimal or completely absent in the mock-injected controls. Surprisingly, EGFP expression was detected in the lens after subretinal injection of AC-GFP but only nominal levels were achieved with injection of TFA-GFP. This finding may also be indicative of the amount of reflux into the vitreous during the subretinal injection procedure as the standard deviation associated with this measurement is quite high.

**Figure 5:**
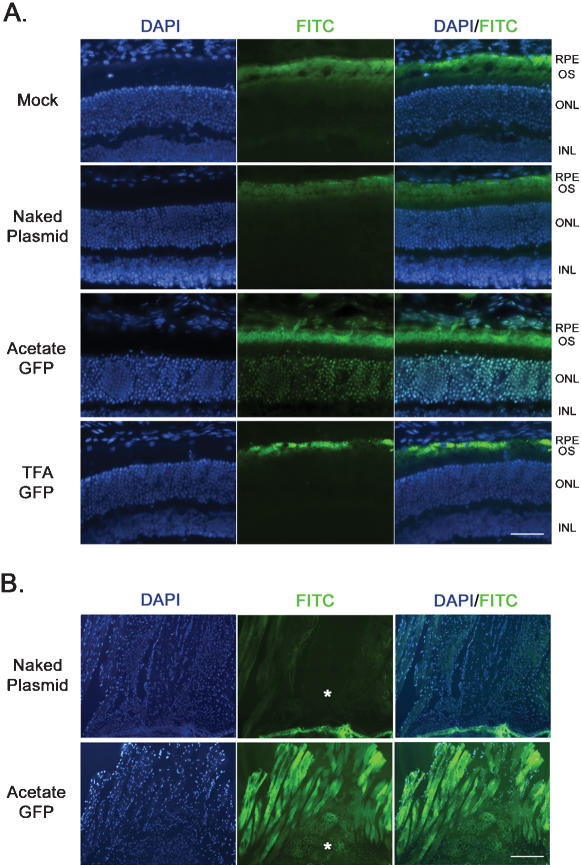
EGFP localization in ocular tissues following subretinal injection. Direct fluorescence microscopy was utilized to localize EGFP expression in ocular tissues at 2-days post-injection of 0.6 µg of compacted DNA. (**A**) EGFP fluorescence was detected in the ONL and RPE of eyes injected with the AC-GFP and TFA-GFP nanoparticles, respectively. (**B**) EGFP was also detected within the optic nerve (asterisks) and the extraocular muscles following injection of the AC-GFP nanoparticle. Scale bars, 10 µM.

Direct fluorescence microscopy was performed on cryosectioned eyes after 2-days post-injection to localize expression throughout the retina. As in the intravitreal samples, retinal autofluorescence was detected throughout the OS layer in both mock and naked plasmid injected samples (Fig 5a). Eyes injected with AC-GFP displayed EGFP expression throughout the outer nuclear layer (ONL), indicative of the uptake and expression of the nanoparticle-delivered plasmid to the photoreceptors. These sections also showed nominal levels of EGFP fluorescence in the RPE and choroid. The TFA-GFP nanoparticles appeared to confer substantial levels of EGFP expression throughout the RPE and sparse expression in the ONL. In several cryosections of eyes subretinally injected with AC-GFP, EGFP fluorescence was observed throughout the optic nerve (Fig 5b). However no fluorescence was detected in brain sections following subretinal injection and this expression was not observed in TFA-GFP injected eyes (data not shown).

**Figure 6:**
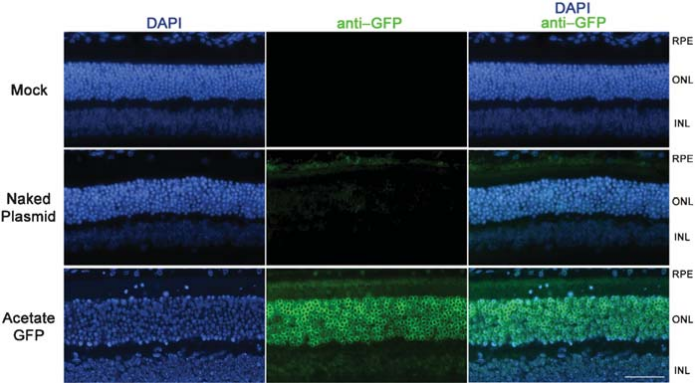
EGFP immunoreactivity following subretinal injection. To eliminate any artifacts due to retinal autofluorescence, immunohistochemistry was performed with a Cy-3-anti-GFP antibody on paraffin-embedded ocular sections from eyes taken at 2-days post-injection of 0.6 µg of compacted DNA. An absence of or minimal EGFP immunoreactivity was found in the mock-injected and naked plasmid-injected eyes, respectively. Examination of sections from eyes injected with the AC-GFP nanoparticle revealed high-level EGFP immunoreactivity in nearly all of the cells in the ONL. Lower levels of EGFP were also detected within the upper half of the INL. Scale bar, 10 µM.

**Figure 7:**
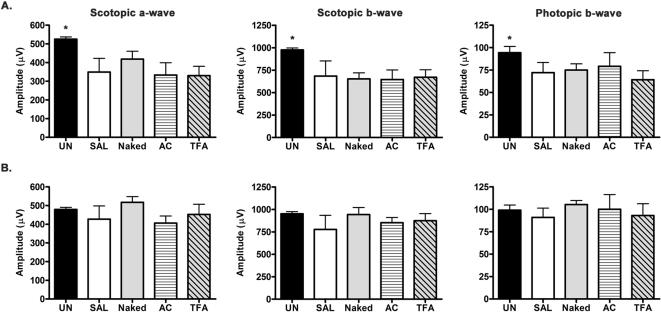
Assessment of retinal function following subretinal injection. ERG analyses were performed in adult Balb/c mice (n = 4) after 7 and 30 days following subretinal delivery of 1 uL saline, naked plasmid, AC-GFP, or TFA-GFP and compared to uninjected controls (UN). To assess rod photoreceptor function and signal transmission **s**cotopic ERG was performed on dark-adapted mice and photopic ERG was performed light-adapted mice to assess cone photoreceptor function. **(A)** At 7 days post-injection, a significant decrease from WT amplitudes was observed in all injected eyes (p<0.05) as an immediate consequence of the injection procedure and subsequent retinal detachment, however there was no significant difference among any of the experimental groups (p>0.05) in the scotopic and photopic amplitudes. **(B)** By 30 days post-injection, no significant difference in scotopic and photopic amplitudes was observed between WT and injected groups (p>0.05), demonstrating the lack of toxicity associated with nanoparticle-transfection in photoreceptors. Significance was determined with ANOVA and Bonferroni's pair-wise comparison test.

To avoid retinal autofluorescence and specifically determine the retinal cell types expressing EGFP after subretinal injection, we performed immunofluorescence microscopy using a Cy-3 conjugated antibody against EGFP (Fig 6). EGFP immunoreactivity was absent in the mock injected controls and showed modest labeling of photoreceptors and RPE cells in the naked plasmid injected samples. However, strong labeling was observed in the AC-GFP injected eyes throughout the entire photoreceptor population. Furthermore, labeling was also present in the RPE and inner nuclear layer (INL). No migration of immune cells into the eye was observed in any sections examined (data not shown). To further determine whether exposure to nanoparticles was toxic to retinal cells, we performed subretinal injections of saline, naked plasmid, AC-GFP, or TFA-GFP and assessed retinal function by electroretinography (ERG) at 7 and 30 days post-injection (Fig 7). If these nanoparticles induced toxicity, the resultant loss of cell viability and number should be apparent when retinal function is assessed. At 7 days post-injection, a reduction in scotopic a- and b-wave as well as in photopic b-wave amplitudes was detected in all samples when compared to uninjected WT mice (Fig 7a). This decrease in retinal function following subretinal injection is consistent with our previous observation and reflects the diminished photransduction potential as a consequence of retinal detachment from the RPE[39]. However, among the injected samples, no significant difference was observed in ERG amplitudes between saline-, naked plasmid-, AC-GFP-, and TFA-GFP-injected samples. By 30 days post-injection, no significant difference in scotopic and photopic amplitudes was detected between WT and any of the injected eyes (Fig 7b). This demonstrates that the compacted DNA nanoparticles have no deleterious effect on the function of photoreceptors or their associated neurons and further confirms the lack of an infiltrating immune response as this would have caused a loss of retinal cells followed by reduction in ERG amplitudes.

**Figure 8:**
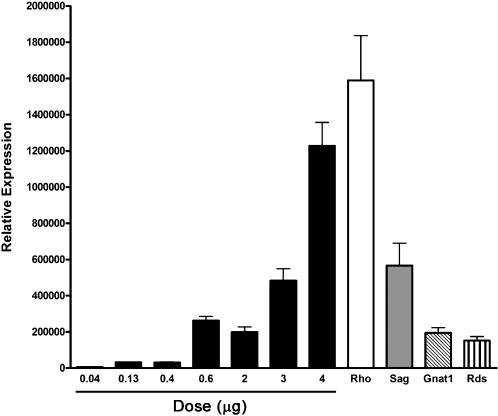
Dose-response of EGFP mRNA levels to increasing amounts of nanoparticle DNA following subretinal injection. Multiple doses of the AC-GFP nanoparticles were diluted in saline and delivered via subretinal injection. qRT-PCR was then performed to assess EGFP mRNA levels in relation to photoreceptor-specific genes. An increasing trend of EGFP mRNA was detected in response to increasing levels of nanoparticle DNA delivered. At the highest dose of 4 µg of DNA, the levels of EGFP were nearly 88% of rod opsin mRNA. Lesser doses achieved expression levels near other photoreceptor-specific genes involved in blinding diseases. Rho: Rhodopsin; Sag: S-Antigen (Arrestin); Gnat1: Guanine Nucleotide Binding Protein, Alpha Transducing 1; Rds: Retinal Degeneration Slow

As different genetic deficits or diseases could require differential levels of gene transfer, we investigated whether nanoparticle transfection could be titrated based upon the dose of DNA delivered. We performed subretinal injection of multiple dilutions of the AC-GFP nanoparticles and assessed retinal expression by qRT-PCR (Fig 8). A distinct correlation was observed between the dose of the nanoparticles injected and the level of retinal transgene expression. We also assessed mRNA levels of several photoreceptor genes that are mutated in human retinal dystrophies to put the levels of EGFP expression into context. We observed that several of the doses tested gave levels of mRNA expression nearly equal to that of the photoreceptor genes examined. These data demonstrate that the dose of compacted DNA nanoparticles can be effectively titrated to obtain desired levels of transgene expression, even comparable to the level of endogenous rhodopsin mRNA.

## Discussion

Herein we have demonstrated that compacted DNA nanoparticles represent a safe and feasible means of gene transfer to various cell types of the eye. High levels of gene expression were documented in the retina, RPE, RGC, INL, and lens and there was no evidence of cellular infiltration or inflammation. We focused on characterizing the effects of gene transfer at 2-days post-injection as this was the peak of reporter gene expression we observed and the CMV promoter used is known to be silenced as early as 1 to 2 days post-transfection [40]–[42]. Although the data here did not examine longevity, other studies our laboratory using different plasmid elements has resulted in an expression duration of several months [43]. Another study demonstrated the efficacy and safety of this methodology in human subjects [32]. This system therefore provides a safe and effective strategy for gene therapy to correct multiple ocular diseases.

Intravitreal injection of the two types of nanoparticles tested drove strong expression of our reporter gene in the inner retina and in ocular tissues near the vitreous. The levels of EGFP mRNA in the lens were relatively equal between eyes receiving the AC-GFP or TFA-GFP nanoparticle, however in the retina and PECS a difference was detected between the two nanoparticles. The TFA-GFP nanoparticle produced EGFP expression levels nearly 5-fold higher than the AC-GFP in the retina. Upon examination of fluorescence from retinal sections, it appears that both nanoparticles conferred expression in RGCs, but only the TFA-GFP produced EGFP within the IPL, suggesting that these particles were able to transfect some cells within the inner nuclear layer. This difference in transfectivity may be explained by the different shapes and sizes of the nanoparticles that are produced by varying the counterion (AC or TFA) at the time of compaction or by different receptor affinities for the two formulations. The AC-GFP nanoparticles consist of rod-shaped structures whereas the TFA-GFP nanoparticles are ellipsoidal in shape (Fig 1). These formulations may have different diffusive properties in the ordered structure of the retina. Additionally, these nanoparticle formulations may have different affinities for the receptor that permits cellular uptake [44], [45]. Varying the counterion has been shown to confer different transfection patterns between cell types. For example, AC but not TFA nanoparticles can transfect striated muscle cells in mice [31], an observation that correlates well with our finding that AC-GFP, but not TFA-GFP, transfects extraocular muscle fibers (Fig 5b).

Subretinal injection of both types of nanoparticles resulted in substantial transfection of the retina and RPE. The TFA-GFP nanoparticle drove strong expression in the RPE cell layer, although it was not as proficient in promoting expression in the photoreceptors. In contrast, the AC-GFP nanoparticle drove strong levels of expression in the entire photoreceptor cell layer and EGFP also was detected in the inner nuclear layer cells nearest to the photoreceptors. Furthermore, the delivery of compacted DNA nanoparticles did not produce a decrease in retinal function as assessed by ERG analyses (Fig 7) and there was no evidence of inflammation in histologic sections of the retina, demonstrating that this methodology does not have any overtly damaging consequences to retinal cells.

Taken together, our data suggest that this mode of gene therapy is applicable for multiple forms of ocular diseases. As intravitreal injection targets the tissues in the front of the eye, this mode of therapy could be widely applicable for corneal diseases such as cataracts and keratoconus. Expression of inflammatory regulators and siRNA may also be effective modes of treating infectious diseases affecting the cornea [46], [47]. This route of injection also was effective in transfecting retinal ganglion cells whereas optic nerve cells were transfected following subretinal injection. The latter finding indicates that AC nanoparticles can be transported in a retrograde fashion to the cell nuclei of optic nerve fibers in the lateral geniculate nucleus. Therefore, these methodologies might be suitable for treating multiple optic nerve diseases, including optic neuritis, Leber's hereditary optic neuropathy, and glaucoma [48], [49]. Specifically, delivery of brain derived neurotrophic factor (BDNF) had a protective effect in animal models of glaucoma [50]. With the use of nanoparticle-mediated gene transfer, it may be possible to have retinal ganglion and optic nerve cells produce substantial levels of BDNF to promote their own sustainability during the stress from intraocular pressure that is observed in glaucoma. For other types of optic nerve injuries, the production of oncomodulin by DNA nanoparticles could be a practical approach for regenerating damaged axons [51]. Also, a recent study demonstrated that ectopic expression of a microbial-type rhodopsin in retinal ganglion cells was capable of restoring visual function in mice lacking photoreceptors [52]. Consequently, intravitreal injection to deliver this gene to RGCs may be a feasible approach for a multitude of retinal degenerative disorders. Furthermore, previous studies using polystyrene nanospheres described the vitreous as a barrier to gene transfer [53], however the specialized compaction procedure utilized for the DNA nanoparticles used here produced a significantly smaller particle that seemed to be freely diffusible through the vitreous.

The subretinal injection showed a dramatic transfection of photoreceptor and RPE cells, demonstrating a significant utility for this non-viral system in rescuing multiple forms of retinal disease. As this system is capable of delivering large DNA cassettes, it would be possible to deliver the entire gene structure in some cases. For many inherited retinal diseases such as retinitis pigmentosa and Stargardt's disease, the disease pathogenesis arising from genetic mutations is understood and gene therapy strategies have already been developed [4], [11], [15], [54]. The development of other complex diseases such as age related macular degeneration (AMD) is not completely understood, although genetic mutations have been identified as risk factors and supplementation of the wild-type genes may be a treatment option [55]–[58]. Current therapies available for the treatment of the “wet” form of AMD involve the use of small molecules to block the activity of vascular endothelial growth factor, but entail recurring injections to maintain this inhibitory effect [59]–[61]. The use of gene transfer to deliver an expression cassette to photoreceptors and RPE cells that produces a similar inhibitory molecule may be a less invasive strategy as it could produce a more sustained effect. This nanoparticle system shows tremendous promise as the basis for developing strategies to treat various diseases of the eye.
